# Seroprevalence and risk factors for peste des petits ruminants and selected differential diagnosis in sheep and goats in Tanzania

**DOI:** 10.1080/20008686.2017.1368336

**Published:** 2017-09-08

**Authors:** Emeli Torsson, Mikael Berg, Gerald Misinzo, Ida Herbe, Tebogo Kgotlele, Malin Päärni, Nils Roos, Anne-Lie Blomström, Karl Ståhl, Jonas Johansson Wensman

**Affiliations:** ^a^ Department of Biomedical Sciences & Veterinary Public Health, Swedish University of Agricultural Sciences, Uppsala, Sweden; ^b^ Department of Veterinary Microbiology and Parasitology, Sokoine University of Agriculture, Morogoro, Tanzania; ^c^ Department of Clinical Sciences, Swedish University of Agricultural Sciences, Uppsala, Sweden; ^d^ National Veterinary Institute, Department of Disease Control and Epidemiology, Uppsala, Sweden

**Keywords:** peste des petits ruminants, contagious caprine pleuropneumonia, foot-and-mouth disease, bluetongue, bovine viral diarrhoea, Tanzania

## Abstract

**Introduction:** Livestock husbandry is critical for food security and poverty reduction in a low-income country like Tanzania. Infectious disease is one of the major constraints reducing the productivity in this sector. Peste des petits ruminants (PPR) is one of the most important diseases affecting small ruminants, but other infectious diseases may also be present.

**Objective:** The objective of this study was to determine the seroprevalence and risk factors for exposure to PPR, contagious caprine pleuropneumonia (CCPP), foot-and-mouth disease (FMD), bluetongue (BT), and bovine viral diarrhoea (BVD) in sheep and goats in Tanzania.

**Methods:** Serum samples were collected in 2014 and 2015, and analysed using enzyme-linked immunosorbent assays to detect antibodies to the five pathogens.

**Results and discussion:** This is the first description of seroprevalence of FMD and BT among small ruminants in Tanzania. Risk factor analysis identified sex (female) (OR for 2014: PPR: 2.49, CCPP: 3.11, FMD: 2.98, BT: 12.4, OR for 2015: PPR: 14.1, CCPP: 1.10, FMD: 2.67, BT: 1.90, BVD: 4.73) and increasing age (>2 years) (OR for 2014: PPR: 14.9, CCPP: 2.34, FMD: 7.52, BT: 126, OR for 2015: PPR: 8.13, CCPP: 1.11, FMD: 2.98, BT: 7.83, BVD: 4.74) as risk factors for exposure to these diseases.

## Introduction

Small ruminants play an important role in food security and livelihood resilience in many parts of the world [1], but there are several constraints reducing the productivity in this sector [2,3]. Infectious disease is considered a major restriction causing direct losses, such as death and decreased production, and indirect losses, such as export constraints [3].

Peste des petits ruminants (PPR) is one of the most important diseases affecting small ruminants worldwide [4,5]. PPR is caused by peste des petits ruminants virus (PPRV), a highly contagious virus that gives rise to disease in sheep, goats, and camels and has also been reported in wild ruminants [6]. Clinical signs of PPR include pyrexia (40–41°C), ocular and nasal discharges, lesions in the oral and nasal mucus membranes, dyspnoea, cough, pneumonia, diarrhoea, and severe dehydration [7]. Morbidity and case fatality rates vary and, depending on factors such as immune status, age, species, and presence of other co-infections, they can be as high as 90–100% [8].

Clinical presentation of PPR can be difficult to differentiate from other diseases affecting small ruminants [7]. Differential diagnoses include contagious caprine pleuropneumonia (CCPP), foot-and-mouth disease (FMD), and bluetongue (BT) [9]. CCPP is caused by the bacterium *Mycoplasma capricolum* subsp. *capripneumoniae* (Mccp) [10], FMD is caused by foot-and-mouth disease virus (FMDV) [11], and BT is caused by bluetongue virus (BTV) and is spread by the vector *Culicoides* mosquitos [12]. Infection with bovine viral diarrhoea virus (BVDV), or the closely related border disease virus (BDV), is generally not considered a differential diagnosis of PPR as these viruses mostly cause reproductive disease in small ruminants [13–15]. However, co-infections with PPRV and BVDV, BDV, or BTV are believed to exacerbate the clinical signs of PPR [16,17].

PPR, CCPP, FMD, and BT are among the 10 most important diseases in sheep and goats worldwide in terms of lost livestock units [5]. For PPR, 6 of the 10 most affected countries during 2006–2009 were African countries [5]. Tanzania, located on the east coast of Africa, is a low-income country with 28.2% of the population living below the national poverty line [18]. Of the total population, 68.4% live in rural areas and three of five rural households earn, on average, 22% of their income from livestock husbandry [19]. Poorer households tend to keep small livestock, such as chicken, sheep and goats, whereas wealthier households keep large livestock [19]. Small ruminants are kept by 52% of Tanzanian households, with an estimated number of 15 million goats and 6 million sheep [20]. PPR was first confirmed in Tanzania in 2008 [21], but a retrospective study on samples collected in the northern districts found antibodies to PPRV were probably already present in 2004 [22]. The disease has since spread to the southern parts of the country and is now considered endemic in the domestic, small ruminant population in the whole country [23–25]. CCPP, FMD, BT, and BVD are endemic in Tanzania [24], however studies on FMD and BT have only been performed on large ruminants.

All of the diseases in question (PPR, CCPP, FMD, BT, and BVD) have been described in wildlife [26–30]. Wild ruminants have been shown to carry PPRV and several species can develop clinical signs of PPR [28,31,32]. Whether interaction or proximity between livestock and wildlife in general, and wild ruminants in particular, is an important risk factor for exposure to PPRV has not yet been determined.

The objective of this study was to estimate the seroprevalence of, and determine possible risk factors for exposure to, PPR, CCPP, FMD, BT, and BVD in small domestic ruminants in selected areas of Tanzania.

## Materials and methods

### Study area and study design

**Figure 1.**
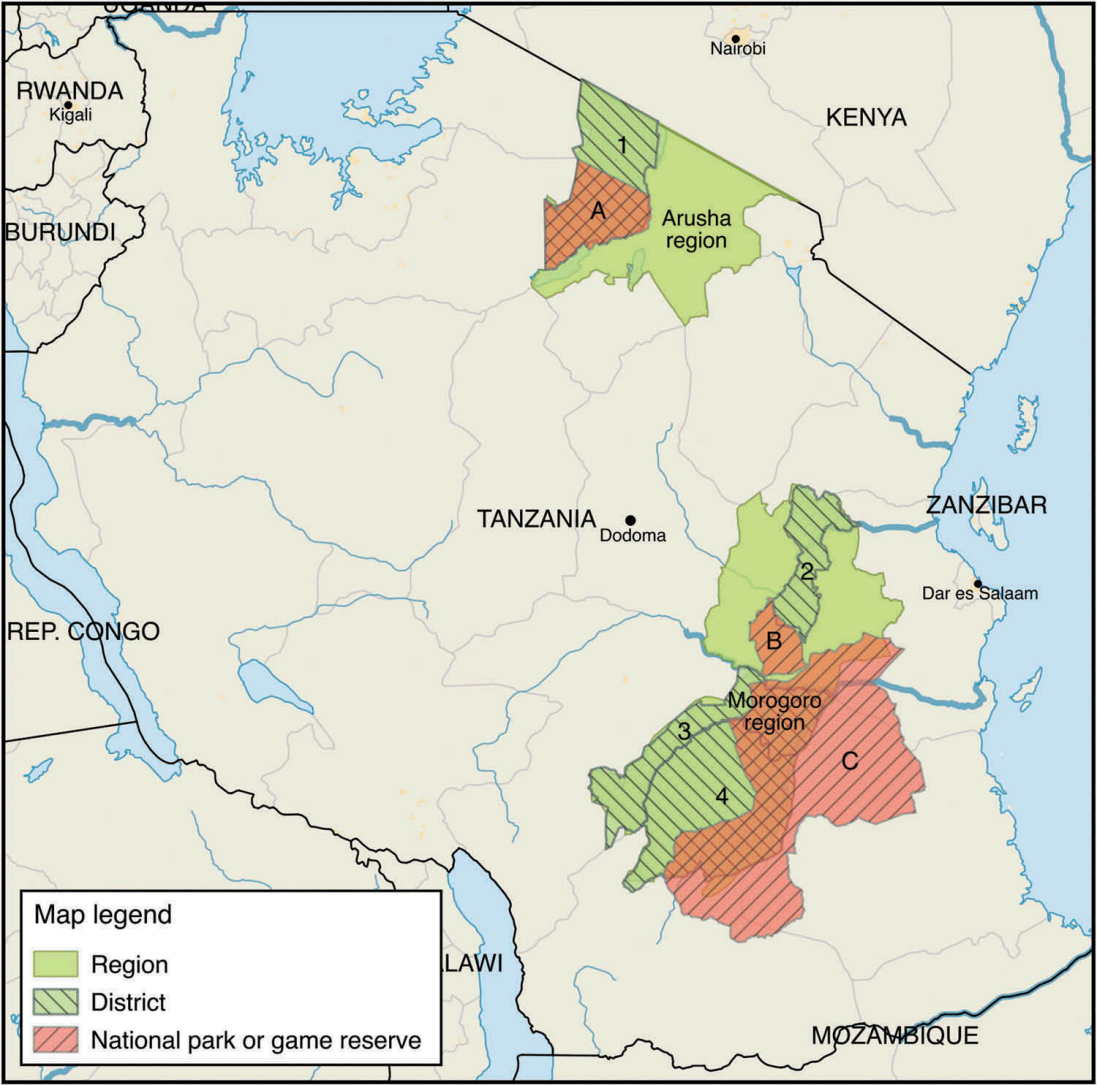
Geographical map of sampling area. Striped green areas indicate visited districts (1 = Ngorongoro, 2 = Mvomero, 3 = Kilombero, 4 = Ulanga). Striped red areas indicate parks or game reserves, i.e. areas with a higher concentration of wildlife (A = Ngorongoro Conservation Area, including Ngorongoro National Park, B = Mikumi National Park, C = Selous Game Reserve).

This study was carried out with the aim to understand the epidemiology of PPR at the wildlife–livestock interface in Tanzania. Thus, the study area was in parts of the country with such an interface (shared pastures, shared water, and regular proximity) and in regions where PPR had previously been described [21,33]. Tanzania is divided into 26 administrative regions, subdivided into districts, and further into wards [34]. Four districts were purposively selected for this study: Ngorongoro in the northern Arusha region, and Ulanga, Kilombero, and Mvomero in the south-eastern Morogoro region (Figure 1). Wards in the districts, located outside, bordering, or within parks or reserves (with a wildlife–livestock interface) were purposively selected, after which 50% were then randomly assigned to the study. In collaboration with local extension officers, wards were replaced with neighbouring wards when those selected did not have enough animals or were inaccessible.

A confidence interval of 95%, a margin of error of 5%, an infinite population, an assumed true overall prevalence of 50% to obtain maximum sample size, and the sensitivity (94.5%) and specificity (99.4%) of the PPR competitive enzyme-linked immunosorbent assay (cELISA) [35] were used [36,37] in calculations of the sample size (PPR cELISA was used to calculate sample size as PPR was the main focus of the study). This gave the needed sample size of 435 samples for each of the two years when herds (containing sheep and/or goats) were visited. To reach the estimated sample size and to assure an even dispersion of the samples in the selected area, we aimed to sample 3 villages in each ward, 2–3 herds per village and 12–20 animals per herd, depending on herd size. If herds were smaller than 12 animals, all of the animals in the herd were sampled.

The study was conducted during two successive years: 2014 (Ngorongoro, Mvomero, and Ulanga) and 2015 (Ulanga and Kilombero). No herd was visited and sampled in both 2014 and 2015. Animals of all ages (2014) were sampled as previously described [38]. However, according to the interview study from this first visit, 43.7% of the sampled animals had been vaccinated against PPRV, possibly resulting in biased prevalence estimates. Therefore, in 2015, young animals (3–12 months) were selected to avoid false positive results due to vaccination or maternal antibodies. If herds did not include enough animals within this age range, older animals were sampled to reach the goal of 12–20 animals per herd.

### Ethical consideration

Sampling was done in collaboration with Tanzania District Veterinary Office, and a local veterinarian or veterinary assistant was present at all sampling sites. Ethical approval was sought and received from the Swedish University of Agricultural Sciences Research Animal Council (SLU ua 2017.1.1.1–1881).

### Sample and data collection

Herds of pastoralists or traditional farmers were visited during September–October 2014 and June–July 2015. Oral consent to sample animals was obtained from the herd owners prior to sample collection. Blood was collected from the jugular vein using sterile needles and vacutainer tubes without additives (BD vacutainer, Plymouth, UK). Blood samples were left to coagulate and separate in a vertical position in a cool box. After separation, the serum was transferred to 2-ml cryotubes and stored at −45°C until analysis.

A pre-prepared questionnaire in English was used for epidemiological data collection at the sampling sites. Interviews were performed in Swahili by a local translator. The questionnaires differed between the two years, due to preliminary results from 2014 and extension of the study for the sample collection in 2015. The 2014 questionnaire included open-ended questions regarding the size of herd, type of animals in the herd, if and when animals were vaccinated against PPR, if the animals interacted with wildlife, and if so, which wildlife species. Interaction with wildlife was specified as physical proximity or shared pastures. The 2015 questionnaire was modified to include information about vaccinations against PPR, CCPP, and FMD, and interaction of the herd with other domestic herds of sheep, goats, or cattle and wildlife. This questionnaire included open-ended questions regarding size of the herd, type of animals in it, if and when animals were vaccinated against PPR, CCPP, and FMD, how often the herd interacted with other domestic herds, latest introduction of new animals into the herd, and how often the herd interacted with wildlife.

### Laboratory analysis

Commercial enzyme-linked immunosorbent assay (ELISA) kits were used to analyse the presence of antibodies to the selected pathogens: *ID screen PPR competition ELISA* (detects anti-PPRV nucleoprotein antibodies [35], sensitivity 94.5%, specificity 99.4%; ID. Vet, Grabels, France), *IDEXX CCPP Ab test* (uses monoclonal antibody ‘4.52’ against *Mycoplasma* sp. Type F38 [39], no information for sensitivity, specificity 99.6%; IDEXX, Hoofddorp, The Netherlands), *ID screen FMD NSP competition* (detects anti-FMDV 3ABC non-structural protein antibodies, sensitivity 100%, specificity 99.4%; ID. Vet, Grabels, France), *Bluetongue Virus (BTV) Antibody Test Kit* (detects anti-BTV VP7 protein antibodies, sensitivity 83%, specificity 100%; IDEXX, Hoofddorp, The Netherlands), and *BVDV p80 Ab Test Kit* (detects anti-BVDV p80 antibodies sensitivity 100%, specificity 99.2%; IDEXX, Hoofddorp, The Netherlands). The *BVDV p80 Ab Test Kit* detects antibodies to both BVDV and BDV, without the ability to differentiate between the two. All kits were used and interpreted according to the manufacturers’ instructions. For PPR, BT, and BVD, there were three different outcomes for the ELISA: positive, negative, or doubtful. In the statistical analysis a doubtful result was considered as negative.

### Statistical analysis

The true prevalence was calculated based on the apparent prevalence and the sensitivity and specificity of the diagnostic tests used, in accordance with [40]. Individual animal results were analysed for possible risk factors for seropositivity, as an indirect measure of exposure: sex, species, age group, and, in the case of PPR and CCPP, vaccination was also included in the analysis, given that the diagnostic test used could not differentiate between infected and vaccinated animals. Age of animal and date of when vaccination had been performed according to the owners were taken into consideration when classifying animals as vaccinated or not. A confidence interval (95%) for the positive proportion was calculated using the score method with continuity correction [41]. To minimize vaccination as a confounder, the animals that owners reported to be vaccinated were excluded from the results in the univariable analysis. All statistical analyses were performed in *R*, Version 3.2.2 [42], and each of the pathogens was analysed separately. Association between risk factors and outcome (i.e. seropositivity to one of the pathogens) was analysed using the command *oddsratio* from the *fmsb* package, with corrections for difference in proportions. A *p*-value < .05 was considered as significant. Risk factors with a *p*-value < .2 in the univariable analysis were analysed in a generalized linear mixed-effect model, using the *glmer* command from the *lme4* package [43]. Risk factors were included as fixed effects, and herd was included as a random effect to account for potential clustering. At herd level in 2014, the risk factors included the district and whether owners reported their animals being in proximity to wildlife, whereas in 2015 they included district, reports of proximity to wildlife, interaction with other domestic herds, and introduction of new animals in the last 12 months. The proportion of positive animals in herds and all risk factors were added to the generalized linear mixed-effect model without previous univaribale analysis. Again, herd was included as a random effect. Interactions between risk factors were tested for in all models.

## Results

### Descriptive analysis

Of 957 animals, 476 animals (from 39 different herds) were sampled in 2014 and 481 animals (from 46 different herds) in 2015. In 2014, 50% of the animals were goats and in 2015 67.2% of the animals were goats. The remainder were sheep. The sex distribution was 73.5% female (including both goats and sheep) in 2014 and 64.9% were female in 2015. In 2014, 17.9% of sampled animals were < 1 year (52.3% females), 26.1% were 1–2 years (58.9% females), and 56% were > 2 years (87.1% females). In 2015, the age distribution was: 53.3% < 1 year (54.3% females), 26.4% 1–2 years (71.4% females), and 20.1% > 2 years (83.3% females).

### Seroprevalence

Antibodies to the pathogens were detected in all visited districts, with some exceptions. BVDV was not detected in the 2014 Ulanga samples, nor was CCPP detected either year in this district. No samples from Ngorongoro were analysed for BT or BVD.

The true prevalence for PPR was estimated at 49.3% (95% CI 44.5;54.0) in 2014 and 10.0% (95% CI 7.1;12.8) in 2015. The true prevalence of FMD was 39.0% (95% CI 33.8;44.3) in 2014 and 14.1% (95% CI 10.9;17.2) in 2015. The highest seroprevalence was for BT for both years: 98.9% (95% CI 90.1;100) in 2014 and 74.5 % (95% CI 68.4;80.6) in 2015. The lowest was for BVD: 3.9% (95% CI 0;8.0) in 2014 and 1.7% (95% CI 0.1;3.4) in 2015. It was not possible to calculate the true prevalence of CCPP because there was no information for sensitivity of the ELISA kit [44]. Observed prevalence for the pathogens is given in Tables 1 and 2.

**Table 1.** Seroprevalence at individual animal level according to sex, species, age group, and vaccination status (PPR) from the first round of a repeated cross-sectional study of small ruminants carried out in Tanzania in 2014.

		PPR		CCPP		FMD		BT		BVD	
Variable		Analysed (positive)	% Positive (95% CI)	Analysed (positive)	% Positive (95% CI)	Analysed (positive)	% Positive (95% CI)	Analysed (positive)	% Positive (95% CI)	Analysed (positive)	% Positive (95% CI)
	Total	476 (223)	46.8 (42.8; 51.4)	323 (47)	14.6 (11.0; 19.0)	340 (134)	39.4 (34.2; 44.8)	106 (87)	82.1 (73.2; 88.6)	106 (5)	4.7 (1.7; 11.2)
Sex	Female	350 (190)	54.3 (48.9; 59.6)	230 (41)	17.8 (13.2; 23.5)	264 (118)	44.7 (38.6; 50.9)	76 (71)	93.4 (84.6; 97.5)	76 (5)	6.6 (2.5; 15.4)
	Male	125 (32)	25.6 (18.4; 34.3)	92 (6)	6.5 (2.7; 14.2)	75 (16)	21.3 (13.0; 32.6)	30 (16)	53.3 (34.6; 71.2)	30 (0)	0 (0; 14.1)
Species	Sheep	238 (108)	45.5 (39.0; 52.0)	159 (2)	1.3 (0.2; 5.0)	182 (64)	35.2 (28.4; 42.7)	60 (48)	80.0 (67.3; 88.8)	60 (2)	3.3 (0.6; 12.5)
	Goat	238 (115)	48.3 (41.8; 54.8)	164 (45)	27.4 (20.9; 35.0)	158 (70)	44.3 (36.5; 52.4)	46 (39)	84.8 (70.5; 93.2)	46 (3)	6.5 (1.7; 18.9)
Age group	< 1 year	85 (15)	17.6 (10.5; 27.7)	60 (0)	0 (0; 7.5)	60 (8)	13.3 (6.3; 25.1)	17 (6)	35.3 (15.3; 61.4)	17 (0)	0 (0; 22.9)
	1–2 years	124 (24)	19.4 (13.1; 27.7)	82 (13)	15.9 (9.1; 26.0)	69 (13)	18.8 (10.8; 30.4)	17 (10)	58.8 (33.4; 80.6)	17 (0)	0 (0; 22.9)
	> 2 years	262 (180)	68.7 (62.7; 74.2)	178 (34)	19.1 (13.8; 25.8)	207 (111)	53.6 (46.6; 60.5)	70 (69)	98.6 (91.3; 99.9)	70 (5)	7.1 (2.6; 16.5)
Vaccination	Yes	208 (131)	63.0 (56.0; 69.5)	–	–	–	–	–	–	–	–
	No	253 (86)	34.0 (28.3; 40.2)	–	–	–	–	–	–	–	–

**Table 2.** Seroprevalence at individual animal level according to sex, species, age group, and vaccination status (PPR and CCPP) from the second round of a repeated cross-sectional study of small ruminants carried out in Tanzania in 2015.

		PPR		CCPP		FMD		BT		BVD	
Variable		Analysed (positive)	% Positive (95% CI)	Analysed (positive)	% Positive (95% CI)	Analysed (positive)	% Positive (95% CI)	Analysed (positive)	% Positive (95% CI)	Analysed (positive)	% Positive (95% CI)
	Total	481 (48)	10.0 (7.5; 13.1)	340 (64)	18.8 (14.9; 23.5)	480 (70)	14.6 (11.6; 18.2)	359 (222)	61.8 (56.5; 66.8)	357 (9)	2.5 (1.2; 4.9)
Sex	Female	312 (45)	14.4 (10.8; 18.9)	217 (42)	19.4 (14.5; 25.4)	312 (57)	18.3 (14.3; 23.1)	223 (156)	70.0 (63.5; 75.8)	223 (8)	3.6 (1.7; 7.2)
	Male	169 (3)	1.8 (0.5; 5.5)	123 (22)	17.9 (11.8; 26.1)	168 (13)	7.7 (4.3; 13.1)	120 (66)	55.0 (45.7; 64.0)	128 (1)	0.8 (0.04; 4.9)
Species	Sheep	158 (13)	8.2 (4.6; 13.9)	92 (3)	3.3 (0.9; 10.0)	157 (15)	9.6 (5.7; 15.6)	96 (51)	53.1 (42.7; 63.3)	97 (4)	4.1 (1.3; 10.8)
	Goat	323 (35)	10.8 (7.7; 14.8)	248 (61)	24.6 (19.5; 30.5)	379 (57)	15.0 (11.6; 19.1)	263 (171)	65.0 (58.9; 70.7)	260 (5)	1.9 (0.7; 4.7)
Age group	< 1 year	255 (9)	3.5 (1.7; 6.8)	180 (32)	17.8 (12.7; 24.3)	255 (30)	11.8 (8.2; 16.6)	187 (89)	47.6 (40.3; 55.0)	188 (3)	1.6 (0.4; 5.0)
	1–2 years	126 (16)	12.7 (7.6; 20.1)	90 (19)	21.1 (13.5; 31.2)	126 (13)	10.3 (5.8; 17.3)	96 (66)	68.8 (58.4; 77.7)	96 (1)	1.0 (0.05; 6.4)
	> 2 years	96 (22)	22.9 (15.2; 32.8)	67 (13)	19.4 (11.1; 31.2)	95 (27)	28.4 (19.9; 38.7)	73 (64)	87.7 (77.4; 93.9)	70 (5)	7.1 (2.6; 16.5)
Vaccination	Yes	81 (19)	23.5 (15.1; 34.5)	42 (0)	0 (0; 10.4)	–	–	–	–	–	–
	No	372 (21)	5.6 (3.6; 8.6)	268 (61)	22.8 (18.0; 28.4)	–	–	–	–	–	–

### Risk factor analysis

Univariable analysis showed a significant difference between male and female animals, with females at higher risk of being seropositive for the tested pathogens, except BVD and CCPP in 2015 (Tables 3-7). Goats were found to be at higher risk than the sheep for seropositivity against CCPP in both years (OR 57.2 in 2014 and OR 9.68 in 2015), and FMD (OR 1.94) and BT (OR 1.64) in 2015 (Tables 4-6). Multivariate analysis identified sex (female) as a significant risk factor for all pathogens, except CCPP and BT in 2015 (Tables 3-7). Increased seropositivity in animals older than 2 years was significant for all pathogens in both years, except for CCPP in 2015.

**Table 3.** Univariable and multivariable analyses for risk factors associated with PPR seropositivity at individual animal level and herd level.

Univariable		2014				2015		
		OR	95% CI	*p*-Value		OR	95% CI	*p*-Value
Sex	Male	2.49	1.37;4.54	.**002**	Male	14.1	1.87;106	**<.001**
	Female				Female			
Species	Sheep	1.01	0.60;1.70	.964	Sheep	1.38	0.49;3.88	.535
	Goat				Goat			
Vaccination	No	3.30	2.25;4.85	**<.001**	No	5.12	2.69;10.0	**<.001**
	Yes				Yes			
Age group	< 1 year	Baseline		**<.001**	< 1 year	Baseline		**<.001**
	1–2 years	1.12	0.56;2.29		1–2 years	3.98	1.70;9.27	
	> 2 years	14.9	8.10;27.5		> 2 years	8.13	3.59;18.4	
**Multivariate**								
Sex	Male	Baseline		.**002**	Male	Baseline		.**006**
	Female	2.78	1.48;5.40		Female	6.18	1.95;28.2	
Vaccination					No	Baseline		.774
					Yes	1.22	0.31;4.93	
Age group					< 1 year	Baseline		
					1–2 years	3.51	1.11;12.4	.**037**
					> 2 years	17.6	3.78;113	**<.001**
Vaccination *Age group	Yes*< 1 year	5.14	1.43;19.3	.**010**				
	Yes*1–2 years	–	–	–				
	Yes*> 2 years	0.86	0.08;10.2	.**010**				
*Herd level*								
District	Ulanga	Baseline			Kilombero	Baseline		
	Mvomero	4.68	2.34;10.5	**<.001**	Ulanga	1.10	0.33;3.72	.874
	Ngorongoro	2.21	0.74;7.27	.155				
Interaction with wildlife		0.59	0.21;1.59	.285	Interaction with wildlife	0.94	0.29;2.75	.910
					Interaction with domestic herds	1.65	0.42;7.71	.476
					Introduction of new animals	1.24	0.31;4.90	.740

Serological results from a repeated cross-sectional study of small ruminants carried out in Tanzania. Factors with *p* < .2 in univariabale analysis were used in multivariate analysis. *p*-Values <.05 were considered significant and are in bold. Interaction between vaccination and age group in samples from 2014 are marked with *.

**Table 4.** Univariable and multivariable analyses for risk factors associated with CCPP seropositivity at individual animal level and herd level.

Univariable		2014				2015		
		OR	95% CI	*p*-Value		OR	95% CI	*p*-Value
Sex	Male	3.11	1.22;7.60	.**010**	Male	1.10	0.62;1.95	.740
	Female				Female			
Species	Sheep	57.2	7.78;420	**<.001**	Sheep	9.68	2.96;31.7	**<.001**
	Goat				Goat			
Age group	< 2 years	2.34	1.18;4.63	.**013**	< 1 year	Baseline		.801
	> 2 years				1–2 years	1.24	0.66;2.33	
					> 2 years	1.11	0.54;2.28	
**Multivariate**								
Sex	Male	Baseline		.**029**				
	Female	4.46	1.24;19.0					
Species	Sheep	Baseline	–	**<.001**	Sheep	Baseline		.**021**
	Goat	81.9	17.4;726		Goat	9.21	1.70;84.0	
Age group	< 2 years	Baseline	–	.**012**				
	> 2 years	5.17	1.54;21.1					
*Herd level*								
Interaction with wildlife		0.60	0.06;4.44	.598	Interaction with wildlife	0.008	<0.01;0.16	.**006**
					Interaction with domestic herds	0.045	<0.01;0.48	.**016**
					Introduction of new animals	4.24	0.14;303	.414

Serological results from a repeated cross-sectional study of small ruminants carried out in Tanzania. Factors with *p* < .2 in univariabale analysis were used in multivariate analysis. *p*-Values <.05 were considered significant and are in bold text.

Analysis at herd level showed a significant association between the Mvomero region (visited in 2014) and seropositivity for PPR and FMD. For FMD, an association with the region Kilombero was significant in 2015 (Tables 3 and 5). Proximity to wildlife was not identified as a risk factor for any of the pathogens for either of the years (Tables 3-7). Rather, proximity to wildlife was identified in 2015 to have a negative association with seropositivity for CCPP. Interaction with other domestic herds was identified to have the same association for CCPP (Table 4). Interaction with other domestic herds was a significant risk factor for being seropositive for FMD and BT in 2015 (Tables 5 and 6).

**Table 5.** Univariable and multivariable analysis for risk factors associated with FMD seropositivity at individual animal level and herd level.

Univariable		2014				2015		
		OR	95% CI	*p*-Value		OR	95% CI	*p*-Value
Sex	Male	2.98	1.63;5.45	**<.001**	Male	2.67	1.41;5.03	.**002**
	Female				Female			
Species	Sheep	1.47	0.95;2.27	.086	Sheep	1.94	1.06;3.56	.**030**
	Goat				Goat			
Age group	< 1 year	Baseline		**<.001**	< 1 year	Baseline		**<.001**
	1–2 years	1.51	0.58;3.93		1–2 years	0.86	0.43:1.72	
	> 2 years	7.52	3.40;16.6		> 2 years	2.98	1.66;5.35	
**Multivariate**								
Sex	Male	Baseline		.**003**	Male	Baseline		.**001**
	Female	3.77	1.58;9.48		Female	4.70	1.91;13.1	
Species	Sheep	Baseline	–	.099	Sheep	Baseline		.**008**
	Goat	1.81	0.90;3.68		Goat	4.19	1.54;13.0	
Age group	< 1 year	Baseline			< 1 year	Baseline		
	1–2 years	1.51	0.4;5.56	.534	1–2 years	1.21	0.46;3.20	.698
	> 2 years	8.73	2.82;30.5	**<.001**	> 2 years	9.10	3.10;30.8	**<.001**
*Herd level*								
District	Ulanga	Baseline			Ulanga	Baseline		.**044**
	Mvomero	25.1	11.1;73.8	**<.001**	Kilombero	6.15	1.02;44.7	
	Ngorongoro	2.36	0.43;12.2	.298				
Interaction with wildlife		1.13	0.36;3.47	.816	Interaction with wildlife	1.52	0.28;8.97	.612
					Interaction with domestic herds	20.7	3.10;262	.**005**
					Introduction of new animals	0.13	0.01;1.18	.**067**

Serological results from a repeated cross-sectional study of small ruminants carried out in Tanzania. Factors with *p* < .2 in univariabale analysis were used in multivariate analysis. *p*-Values <.05 were considered significant and are in bold text.

**Table 6.** Univariable and multivariable analyses for risk factors associated with BT seropositivity at individual animal level and herd level.

Univariable		2014				2015		
		OR	95% CI	*p*-Value		OR	95% CI	*p*-Value
Sex	Male	12.4	3.90;39.5	**<.001**	Male	1.90	1.22;2.95	.**004**
	Female				Female			
Species	Sheep	1.39	0.50;3.88	.527	Sheep	1.64	1.02;2.64	.**040**
	Goat				Goat			
Age group	< 1 year	Baseline		**<.001**	< 1 year	Baseline		**<.001**
	1–2 years	2.62	0.65;10.5		1–2 years	2.42	1.44;4.07	
	> 2 years	126	13.9;1153		> 2 years	7.83	3.68;16.7	
**Multivariate**								
Sex	Male	Baseline	–	.**030**	Male	Baseline		.437
	Female	7.49	1.29;63.9		Female	1.26	0.70;2.27	
Species					Sheep	Baseline		.**023**
					Goat	2.32	1.14;4.93	
Age group	< 1 year	Baseline	–	–	< 1 year	Baseline		
	1–2 years	3.34	0.42;155	.319	1–2 years	3.04	1.53;6.32	.**002**
	> 2 years	183	15.2;23,216	.**001**	> 2 years	18.4	6.61;61.4	**<.001**
*Herd level*								
District	Ulanga	Baseline		.356				
	Mvomero	2.42	0.30;21.9					
Interaction with wildlife		0.57	0.03;7.61	.636	Interaction with wildlife	1.18	0.48;2.94	.698
					Interaction with domestic herds	3.85	1.55;10.5	.**004**
					Introduction of new animals	0.99	0.26;3.65	.983

Serological results from a repeated cross-sectional study of small ruminants carried out in Tanzania. Factors with *p* < .2 in univariabale analysis were used in multivariate analysis. *p*-Values <.05 were considered significant and are in bold text.

**Table 7.** Univariable analysis for risk factor associated with BVD seropositivity at individual animal level.

Univariable		2014				2015		
		OR	95% CI	*p*-Value		OR	95% CI	*p*-Value
Sex	Male	–	–	.150	Male	4.73	0.58;38.2	.110
	Female				Female			
Species	Sheep	2.02	0.32;12.6	.445	Sheep	0.46	0.12;1.73	.239
	Goat				Goat			
Age group	< 1 year	–	–	.279	< 1 year	Baseline		.**023**
	1–2 years				1–2 years	0.65	0.07;6.33	
	> 2 years				> 2 years	4.74	1.10;20.4	

Serological results from a repeated cross-sectional study of small ruminants carried out in Tanzania. There were no positive male animals or age groups <1 and 1–2 years in samples from 2014, so it was not possible to obtain OR for the risk factor ‘age group’ or ‘sex’. Multivariate analysis was not possible due to an insufficient number of seropositive animals. *p*-Values <.05 were considered significant and are in bold text.

An interaction was found between the variables *age group* and *vaccination against PPR* in the samples from 2014. Effect of vaccination against PPR differed among the age groups.

## Discussion

In this study, we investigated the seroprevalence of PPR and some of its differential diagnoses in selected areas in Tanzania. Commercial ELISA tests were used to detect antibodies in serum samples from sheep and goats. The serological results were used further to calculate risk factors for exposure to PPRV, Mccp, FMDV, BTV, and BVDV. In Tanzania, and other east African countries, small ruminant production is an important livelihood for a significant proportion of the population [19]. This important position of small ruminants is one of the reasons behind the joint Food and Agriculture Organization (FAO) and World Organization for Animal Health programme to control and eradicate PPR and control small ruminant diseases [4]. PPRV is quickly increasing its spread across the world and is now threatening the most southern countries of Africa, with Tanzania currently being its southern border on the east coast [25]. To stop the spread further south, it is important to understand the prevalence and epidemiology of both PPR and its most common differential diagnosis, as the clinical presentation can be difficult to diagnose [4].

The calculated true seroprevalence for PPR was 49.3% in 2014 and 10.0% in 2015. A vaccination campaign had been carried out in the Morogoro and Mtwara region prior to our sample collection [45], which may have influenced the 2014 results. Therefore, we aimed to sample animals aged 3–12 months in 2015, as animals in this age group would not have been alive during the vaccination campaign. As expected for an endemic disease, where survival of infection results in lifelong immunity, age was identified as a risk factor for exposure (Table 3). Age and vaccination bias of sampled animals could be the reasons for the difference in seroprevalence in 2014 and 2015. In addition, we did not visit the same areas both years; the differences in seroprevalence could therefore have been due to geographical differences. Previous studies in northern Tanzania found an overall seroprevalence of 45.5% in 2008 [46] and 22.1% in 2008–2009 [21]. In southern Tanzania, in the Mtwara region bordering Mozambique, 31% of sampled small ruminants had antibodies to PPRV [47]. A recent study analysing samples from 14 different regions of Tanzania described an overall seroprevalence of 27.1%, with regions varying from 2.4% (Kagera) to 72.8% (Morogoro), demonstrating the varying level of seroprevalence within the country [23].

Sex has previously been described as a risk factor for PPR; mostly females are identified to be at higher risk [21,48,49]. However, some studies found the opposite association [50–52]. Our results suggest that females had a higher risk of being seropositive for PPRV in both 2014 and 2015. Previous studies on risk factors for PPR have suggested that females are kept longer by their owners (to be used in reproduction), and therefore have a longer risk period for PPRV exposure [48]. In addition, females are more likely to be vaccinated, which may bias the results. The stress associated with pregnancy and milk production may also predispose females to infection [48,49]. Differences between the studies, such as management systems or breed of sheep and goats, may also influence the results. In our study, we found that the age group *>*2* *years was mainly composed of females. This age group had the highest proportion of seropositive individuals; the result might be due to a selection bias. However, the multivariable analysis did not find an interaction between these two variables, indicating that this cannot be the entire explanation.

The true prevalence for CCPP was not possible to calculate because there was no available information on sensitivity for the ELISA test used [44]; however, the apparent prevalence was 14.6% in 2014 and 18.8% in 2015. Previously, a prevalence of 51.2% (in 2007) and 33.7% (in 2009) had been described in southern Tanzania [51].

Goats were identified to be at higher risk than sheep for seropositivity towards CCPP in both years, due to CCPP having a higher affinity for goats. Sheep can develop clinical signs following infection by CCPP, but the infection can also be subclinical [53].

The calculated true prevalence for FMD was 39.0% in 2014 and 14.1% in 2015 for both sheep and goats. To the best of our knowledge, no previous reports of seroprevalence of FMD in small ruminants in Tanzania are available. FMDV causes a less severe disease in small ruminants compared with large ruminants [54]; however, the oral lesions sometimes seen even in small ruminants make FMD an important differential diagnosis of PPR, especially in light of the attempt to eradicate PPR [7]. A study in neighbouring Uganda found a seroprevalence of 14% in goats and 22% in sheep [55]. Seroprevalence of FMD in buffalo and cattle in Tanzania is high. Mkama et al. [56] found an overall prevalence of 76.3% (248 of 330) for buffalo and cattle, with the buffalos from western Tanzania having a 100% seroprevalence (29 of 29). Antibodies to FMDV decrease faster in sheep than cattle [57], which could be one explanation for the difference in seroprevalence between small and large ruminants. As for PPR, our study identified age as a risk factor for FMDV exposure. Age is a documented risk factor for FMDV exposure in cattle, both in endemic and epidemic settings [58,59]. A higher age gives a longer time to be exposed in the endemic setting, and the higher mortality seen in younger animals leaves the older seropositive animals to be sampled [59,60]. Also in line with the results for PPR, female animals were identified to be at a higher risk than males for FMDV exposure. Similar explanations in PPR can be applied to FMD as well, with the exception of vaccination. None of the owners reported that their animals had been vaccinated against FMD.

The calculated true prevalence for BT was 98.9% in 2014 and 74.5% in 2015. No previous studies have been done on seroprevalence of BT in domestic animals in Tanzania. As with FMD, possible oral lesions caused by BTV makes it an important differential diagnosis of PPR [12]. Free-living wild buffalos from eight different areas in Tanzania were sampled between 1987 and 1989 and analysed for antibodies to a selection of pathogens, including BT [61]. An overall prevalence of 91.6% was found, with six of eight areas having a 100% prevalence [61]. A similar study was performed on wildlife in Zimbabwe with samples collected between 1989 and 1995 [62]. Most samples came from buffaloes, followed by different species of antelopes, and also white and black rhinoceroses. An overall prevalence of antibodies to BTV of 44.1% was found [62]. Domestic cattle were sampled in western Sudan and serological evidence of BTV infection was found in 19.4% of them (58 of 299) [63]. Our results are more in line with those from [59] and [60]. A high seroprevalence is expected from a virus that often gives a subclinical or unapparent disease in ruminants and is spread very efficiently by its vector [12]. Risk factors identified for exposure to BTV included age and sex (Table 6), as for the other pathogens in this study. Age as a risk factor for exposure to BTV is in agreement with a risk factor analysis in cattle in western Sudan [63]. In 2015, multivariate analysis identified goats as being at higher risk for exposure to BTV than sheep. In 2015, 73% of samples analysed came from goats, which may have biased the result.

The calculated true prevalence for BVD was 3.9% in 2014 and 1.7% in 2015. This is lower than what has previously been described for domestic animals in Tanzania. In Tanzanian samples collected between 1985 and 1987 from cattle, sheep, and goats, evidence of BVD exposure was described in 34.0% of cattle, 32.1% of sheep, and 24.9% of goats [64]. In wild buffaloes, mainly from northern parts of Tanzania, 16.9% had antibodies to BVDV [61]. Five cattle herds in the Kafue flats of Zambia were tested for antibodies to a selection of pathogens, and 76.2% were positive for BVDV [65]. A more recent study was performed in western Kenya; calves aged 3–7 days were tested for antibodies to BVDV and an adjusted seroprevalence of 19.8% was identified [66]. Seroprevalence for BVD varies significantly between the different studies, with our study having the lowest prevalence. Dissimilarities in the studies include differences in production of animals sampled, method of analysis, year of sampling, and study design, which makes comparisons difficult. Univariable analysis of our serological results from 2015 identified age (> 2 years) as a risk factor for exposure to BVD. Because of the low number of seropositive animals (9 out of 357), further studies are warranted before making any definite conclusions on risk factors for exposure. Multivariate analysis could not be performed with the BVD results due to too few positive samples.

Correlation between seropositivity for the studied pathogens, except BVD, was analysed at herd level; a generalized linear mixed-effect model was used to identify risk factors affecting the entire herd. In this study no difference was found, for any of the pathogens, between herds with proximity to wildlife and those without. PPRV has long been known to cause disease in wildlife [28]. Clinical signs are yet to be described in wild ruminants in sub-Saharan Africa, but have been reported in wild ruminants in Asia and in the Middle East [67]. Antibodies have been described in wild buffaloes, Grant’s gazelle, wildebeest, and impala in Tanzania [32,68]. Recently, a Grant’s gazelle without clinical signs of PPR in northern Tanzania tested positive on real-time reverse transcription polymerase chain reaction [32]. The gazelle was sampled in an area with an ongoing outbreak of PPR among domestic animals. The same study found a 63% seroprevalence in 46 sampled wild ruminants [32]. Although it is probable that PPR transmits between domestic and wild animals [32,69,70], our results do not support the hypothesis of wildlife as an important risk factor for exposure for domestic animals in an endemic setting. For the closely related rinderpest virus, the well-accepted hypothesis was that infection in wildlife was not self-sustaining, but rather a case of spillover from domestic animals [71,72]. The same hypothesis has been suggested for PPRV [32,73], and our results seem to be in agreement with this.

For FMD, contact with wildlife has been described as an important risk factor for infection in domestic animals in sub-Saharan Africa [74]. However, among wildlife species, only the African buffalo has been identified as a long-term maintenance host [74]. Small ruminants are highly susceptible to FMDV infection, but they are not as efficient as cattle in maintaining the infection within the population [11]. Years of experience with FMD in southern Africa have been unable to reveal small ruminants as an important part of the maintenance or transmission of the disease [75]. Our results did not identify proximity to wildlife as a risk factor for FMD in domestic small ruminants in these areas of Tanzania.

Bluetongue virus is endemic in both the domestic and wild populations of many African countries [12]. Various wildlife species, both in Africa and in Europe, have been discussed as possible reservoirs [62,76,77]. The epidemiology of BTV differs from the other viruses studied here, as it is spread through its vector, the *Culicoides* mosquito, not through direct contact. In parts of Europe where BT is endemic, studies suggest that wild ruminants, mainly red deer, play a role in the epidemiology [76]. Our results did not indicate proximity to wildlife as an important risk factor for small ruminants to be exposed to BTV in the studied area. However, we did identify interaction with other domestic herds as a risk factor, in agreement with a previous study of sheep and goats in Iran [78]. Possibly the vector is attracted by the increased number of animals in the same location.

For CCPP in 2015, proximity to wildlife had a statistically significant negative association, as did interaction with other domestic herds (Table 4). The ELISA used for detection of CCPP is specific for antibodies against Mccp [44]; however, cross-protection between different subspecies of mycoplasmas cannot be excluded [53,79,80]. It is possible that other members of the *Mycoplasma mycoides* cluster are circulating in the studied areas and producing cross-protection against CCPP.

Limitations of this study include none of the ELISAs used being able to differentiate between vaccinated and naturally infected animals, and all questionnaire data being collected by a local translator. Information regarding vaccination status of the animals was acquired from the owners. Owners could, for a variety of reasons, provide incorrect information; for example, they do not remember, or a previous owner had the animals vaccinated. To minimize this bias during the sample collection in 2015, we targeted animals 3–12 months of age, animals the owners were more likely to have the correct information about. Further, we used the information from the questionnaires to study whether interaction with wildlife was a possible risk factor for exposure to the studied pathogens. Owners were asked how often the animals had contact with wildlife. The question could, however, have been misunderstood or interpreted in a different way than what we intended. Answers given to the question were, for example: ‘never’, ‘during dry season’, and ‘everyday’. The interaction between wildlife and livestock can be measured using several methods, with the usage of a questionnaire and local knowledge being a fast and practical method to get preliminary data [81]. The method is not, however, as precise as others, and this insecurity should be considered when interpreting the results of the risk factor analysis.

## Conclusion

This study confirmed the presence of antibodies to PPRV, CCPP, FMDV, BTV, and BVDV in sheep and goats in northern and south-eastern Tanzania, indicating a continuous circulation of these pathogens. This is the first description of the presence of antibodies for FMD and BT in small ruminants in Tanzania. Risk factor analysis at individual animal level identified sex (female) and increasing age as two important factors influencing level of exposure to infection. Proximity to wildlife was not identified as a risk factor for any of the pathogens studied.
